# Functioning in schizophrenia from the perspective of psychologists: A worldwide study

**DOI:** 10.1371/journal.pone.0217936

**Published:** 2019-06-06

**Authors:** Laura Nuño, Georgina Guilera, Michaela Coenen, Emilio Rojo, Juana Gómez-Benito, Maite Barrios

**Affiliations:** 1 Clinical Institute of Neurosciences (ICN), Hospital Clinic, Barcelona, Spain; 2 Department of Social Psychology and Quantitative Psychology, University of Barcelona, Barcelona, Spain; 3 Group on Measurement Invariance and Analysis of Change (GEIMAC), Institute of Neurosciences, University of Barcelona, Barcelona, Spain; 4 Institute for Medical Information Processing, Biometry and Epidemiology–IBE, Research Unit for Biopsychosocial Health, Ludwig-Maximilians-Universität (LMU), Munich, Germany; 5 Pettenkofer School of Public Health, Munich, Germany; 6 ICF Research Branch, a cooperation partner within the WHO Collaborating Centre for the Family of International Classifications in Germany (at DIMDI), Munich, Germany; 7 Hospital Benito Menni CASM, Sisters Hospitallers, Sant Boi de Llobregat, Spain; 8 Department of Psychiatry, International University of Catalonia, Barcelona, Spain; University of La Rioja, SPAIN

## Abstract

Schizophrenia is a severe mental disorder associated with impairment in functioning. A multidisciplinary approach is essential to help individuals with this health condition, and psychological interventions are considered a priority. The International Classification of Functioning, Disability and Health (ICF) offers a theoretical framework for assessing functioning and disability. The ICF Core Sets for schizophrenia are a list of ICF categories describing the most common problems in functioning of persons affected by this health condition. This study aimed to explore the content validity of these ICF Core Sets and to identify the most common problems in people with schizophrenia from the perspective of psychologists. Psychologists with experience of schizophrenia treatment were recruited for a three-round Delphi study in order to gather their views regarding the problems commonly presented by these patients. A total of 175 psychologists from 46 countries covering the six WHO regions answered the first-round questionnaire, and 137 completed all three rounds. The 7,526 concepts extracted from first-round responses were linked to 412 ICF categories and 53 personal factors. Consensus (≥75% agreement) was reached for 76 ICF categories and 28 personal factors. Seventy-three of the 97 ICF categories that form the Comprehensive ICF Core Set for schizophrenia achieved consensus, and only three categories that yielded consensus do not feature in this Core Set. These results support the content validity of these ICF Core Sets from the perspective of psychologists. This provides further evidence of the suitability of the ICF framework for describing functioning and disability in persons with schizophrenia.

## Introduction

Schizophrenia is a severe mental disorder that afflicts more than 21 million people worldwide [1]. It has a multifactorial etiology, with numerous individual variables interacting with several environmental factors [2]. Its lifetime prevalence is estimated at between 0.3% and 0.7%. The disorder is characterized by the presence of delusions, hallucinations, disorganized thinking, abnormal motor behavior (including catatonia), and negative symptoms[3]. Although this wide range of symptoms can be present in different combinations[4], patients across the schizophrenia spectrum commonly experience impairments, limitations, and restrictions in major areas of functioning (such as education, work, interpersonal relations, or self-care). Better and more targeted treatment of these areas would help to decrease the stigma that surrounds this illness and empower patients to improve their quality of life [5].

A multidisciplinary approach to both assessment and clinical intervention is essential to support individuals with this health condition. Worldwide clinical guidelines consider psychological interventions to be one of the mainstays of treatment and emphasize the importance of cognitive-behavioral therapy, cognitive remediation, and family intervention [6–8]. The goals of these interventions are manifold, with key targets being to improve psychological wellbeing and quality of life, neurocognition, and family communication. Other main objectives include training in social skills and problem solving, reducing positive and negative symptoms, and modifying contextual factors to facilitate recovery [9]. Psychological assessment focuses on the same areas and encompasses both neuropsychological testing and the evaluation of psychosocial functioning [10].

**Fig 1:**
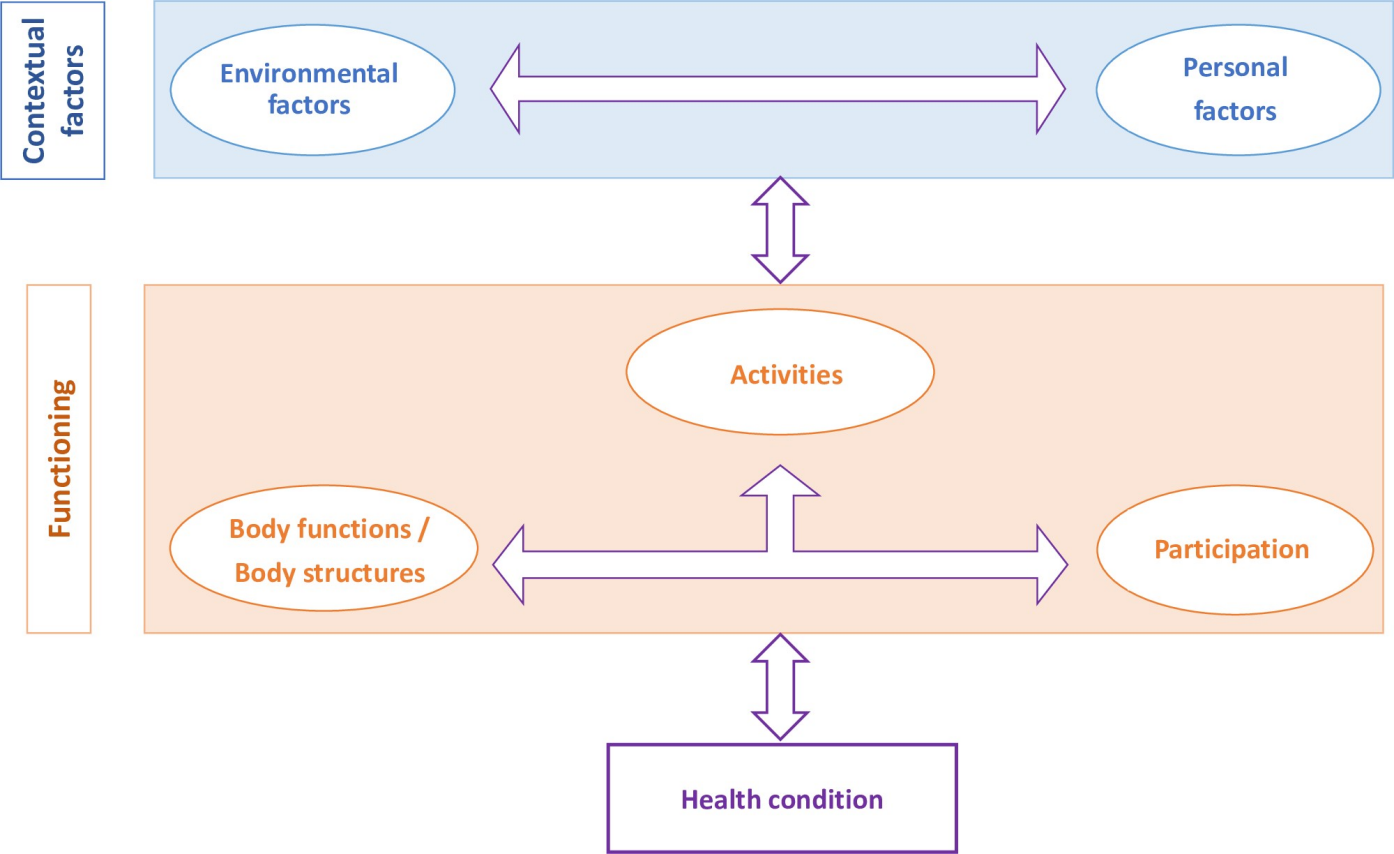
Integrative biopsychosocial model of functioning and disability.

Achieving these therapeutic goals requires a proper understanding of each patient’s functioning and health status. At the 54th World Health Assembly on 22 May 2001 the International Classification of Functioning, Disability and Health (ICF) was officially endorsed (resolution WHA 54.21) by all 191 member states of the World Health Organization (WHO) as the international standard to describe and measure health and disability [11]. The ICF is based on a multidimensional, biopsychosocial approach (see Fig 1) and considers a patient’s functioning as a dynamic interaction between the underlying health condition and specific personal and environmental contextual factors. Its worldwide acceptance and applicability to all health conditions is one of its main contributions in comparison with other evaluation systems. Another key strength is its multidisciplinary approach, insofar as it provides a common language that can be used by all the professionals and healthcare disciplines involved in a person’s care. A comprehensive framework employing a universal language that is understood by all actors could improve the implementation of care plans, leading to a common understanding and shared goals between all health professionals. The ICF provides just such a framework.

The ICF as a whole includes more than 1400 categories and hence is not suited to application in everyday clinical practice. Consequently, the WHO has established a protocol to develop ICF Core Sets (ICF-CSs) for specific health conditions. Each ICF-CS comprises a selection of ICF categories that are considered essential for describing the functioning of a person living with the corresponding health condition. Following the methodology endorsed by the WHO [12], the ICF-CSs for schizophrenia have already been developed through a formal decision-making consensus process, integrating evidence from four preparatory studies and expert opinion [13]. The Comprehensive ICF-CS for schizophrenia consists of 97 categories covering the characteristic spectrum of problems in functioning and health that are experienced by individuals with this disorder; it also includes environmental factors. The Brief ICF-CS for schizophrenia includes just 25 of these categories, the ones considered most important for the purposes of assessment and treatment. The two ICF-CSs for schizophrenia are available for free download at: https://www.icf-research-branch.org/icf-core-sets-projects2/mental-health/icf-core-set-for-schizophrenia.

A basic requirement for the implementation of these ICF-CSs in clinical practice is their validation from different perspectives. The content validity of the ICF-CSs for schizophrenia has already been examined and supported from the perspective of psychiatrists [14]. The goal of the present study was to build on this by exploring content validity from the perspective of psychologists, another group of health professionals closely involved in the care of individuals with schizophrenia. Specifically, our two objectives were: 1) to identify the problems, personal characteristics/resources, and aspects of the environment that psychologists regard as most important for understanding functioning in people with schizophrenia; and 2) to analyze the extent to which the problems and aspects identified are represented in the ICF-CSs for schizophrenia.

## Method

We conducted a three-round worldwide Delphi study by means of an e-mail survey. This is a multistage process in which each stage or round builds on the results of the previous one in order to gather and provide information about a particular subject [15]. The purpose is to achieve consensus from a panel of individuals with knowledge of the topic of interest (hereinafter, experts). The Institutional Review Board Committee of University of Barcelona approved the Study IBR00003099. Participants were provide with a written consent form. The study procedure was the same as that used in the validation study of the ICF-CS for schizophrenia from the perspective of psychiatrists, and hence further details can be consulted in Nuño et al. (2018) [14].

### Recruitment of participants

Expert psychologists from around the world were recruited by contacting international associations of psychologists, universities with health professional training programs, and hospitals. We also made use of literature searches, LinkedIn contacts, and personal recommendations. To ensure that study participants were all “informed individuals” with regard to the treatment of individuals with schizophrenia, the initial invitation letter specified that they should be “psychologists experienced in the treatment of schizophrenia”. In addition, it was made clear that they should have at least one year experience of treating adults with schizophrenia.

Our aim was to recruit a panel of experts as broad and heterogeneous as possible and to achieve consensus and common opinion despite and across this variability. Indeed, we sought to obtain a sample of experts that, as far as possible, reflected worldwide variety in all the variables considered (e.g., gender, age, years of experience, and region). Furthermore, experts did not need to have specific knowledge about the ICF, and they were selected without taking into account their therapeutic orientation or training background. It was made clear that they should base their answers on their clinical experience. Those psychologists who had participated in any earlier stage of developing the ICF-CS for schizophrenia were not eligible for the present study.

All potential participants received an invitation with basic information about the study and what would be required of them. They were also asked to provide demographic and professional data. Of the 1,555 health professionals who agreed to take part and who provided demographic and professional data, 223 were psychologists who met the eligibility criteria and who were therefore invited to begin round one of this study.

A total of 175 psychologists from 46 countries covering the six WHO regions answered the first-round survey (78.5% of the 223 who were sent the survey material). They primarily worked in clinical practice (mean 46.3% of their time), followed by research (28.1%), teaching and training (16.9%), management (7.8%), and other tasks (0.9%). Table 1 shows participants’ demographic and professional characteristics. The second-round survey was answered by 151 psychologists, and 137 completed the third round, with a response rate across rounds one to three of 78.3%.

**Table 1:** Distribution of participants across the three Delphi rounds and demographic and professional data obtained from participants in the first round.

WHO region	Round 1 n (%)	Female n (%)	Age Mean (range)	Experience in schizophrenia [years] Mean (range)	Expertise^a^ Mean (range)	Population treated^b^				Participation based on Round 1	
						Acute n (%)	Chronic n (%)	Rural n (%)	Urban n (%)	Round 2 n (%)	Round 3 n (%)
Africa^c^	11 (6.3)	8 (72.7)	39.45 (31–50)	7 (2–18)	3.3 (2–5)	8 (72.7)	8 (72.7)	5 (45.5)	10 (90.9)	9 (81.8)	9 (81.8)
Americas^d^	47 (26.9)	28 (59.6)	45.0 (28–67)	14.1 (1–42)	3.9 (1–5)	25 (53.2)	44 (93.6)	14 (29.8)	32 (68.1)	41 (87.2)	37 (78.7)
Eastern Mediterranean^e^	21 (12.0)	14 (66.7)	37.3 (24–56)	7.43 (1–23)	3.1 (1–5)	12 (57.1)	15 (71.4)	9 (42.9)	16 (76.2)	12 (57.1)	10 (47.6)
Europe^f^	63 (36.0)	38 (60.3)	43.06 (28–66)	12.8 (2–37)	3.6 (1–5)	30 (47.6)	53 (84.1)	15 (23.8)	49 (77.8)	59 (95.0)	55 (87.3)
South-East Asia^g^	20 (11.4)	13 (65.0)	34.4 (25–51)	7.6 (1–18)	3.3 (2–5)	8 (40.0)	17 (85.0)	12 (60.0)	13 (65.0)	19 (95.0)	15 (75.0)
Western Pacific^h^	13 (7.4)	9 (69.2)	44.7 (32–64)	14.7 (5–30)	4.2 (3–5)	9 (69.2)	12 (92.3)	5 (38.5)	10 (76.9)	11 (84.6)	11 (84.6)
Total	175	110 (62.9)	41.8 (24–67)	11.7 (1–42)	3.6 (1–5)	92 (52.6)	149 (85.1)	60 (34.3)	130 (74.3)	151 (86.3)	137 (78.3)

^a^ Self-rating of schizophrenia expertise: 1 = limited expertise to 5 = extensive expertise.

^b^ It was possible to select more than one option.

^c^ Algeria, Kenya, Mozambique, South Africa, and Zimbabwe.

^d^ Argentina, Brazil, Canada, Chile, Colombia, Cuba, Ecuador, Mexico, and United States of America.

^e^ Egypt, Iran, Jordan, Libya, Pakistan, Saudi Arabia, and United Arab Emirates.

^f^ Cyprus, Czech Republic, Denmark, France, Germany, Greece, Hungary, Ireland, Italy, Macedonia, Netherlands, Norway, Poland, Russia, Spain, Sweden, Turkey, and United Kingdom.

^g^ Bangladesh, India, and Indonesia.

^h^ Australia, China, Japan, and Singapore.

There were no statistically significant differences in age, gender, or population treated (urban, rural, acute, and chronic) between psychologists who responded in the first round and those were invited to take part but did not do so. However, there was a significant difference between these two groups in years of experience (p < .01), since the invited experts who did not respond were less experienced than those who did take part. Specifically, 52% of invited experts who did not respond had less than five years’ experience in the treatment of individuals with schizophrenia, while this was the case for only 20% of the experts who did take part in the first round.

There were no significant differences in age, gender, or years of experience in treating individuals with schizophrenia between the groups that responded across rounds 1 to 3.

### Material and data collection

**Fig 2:**
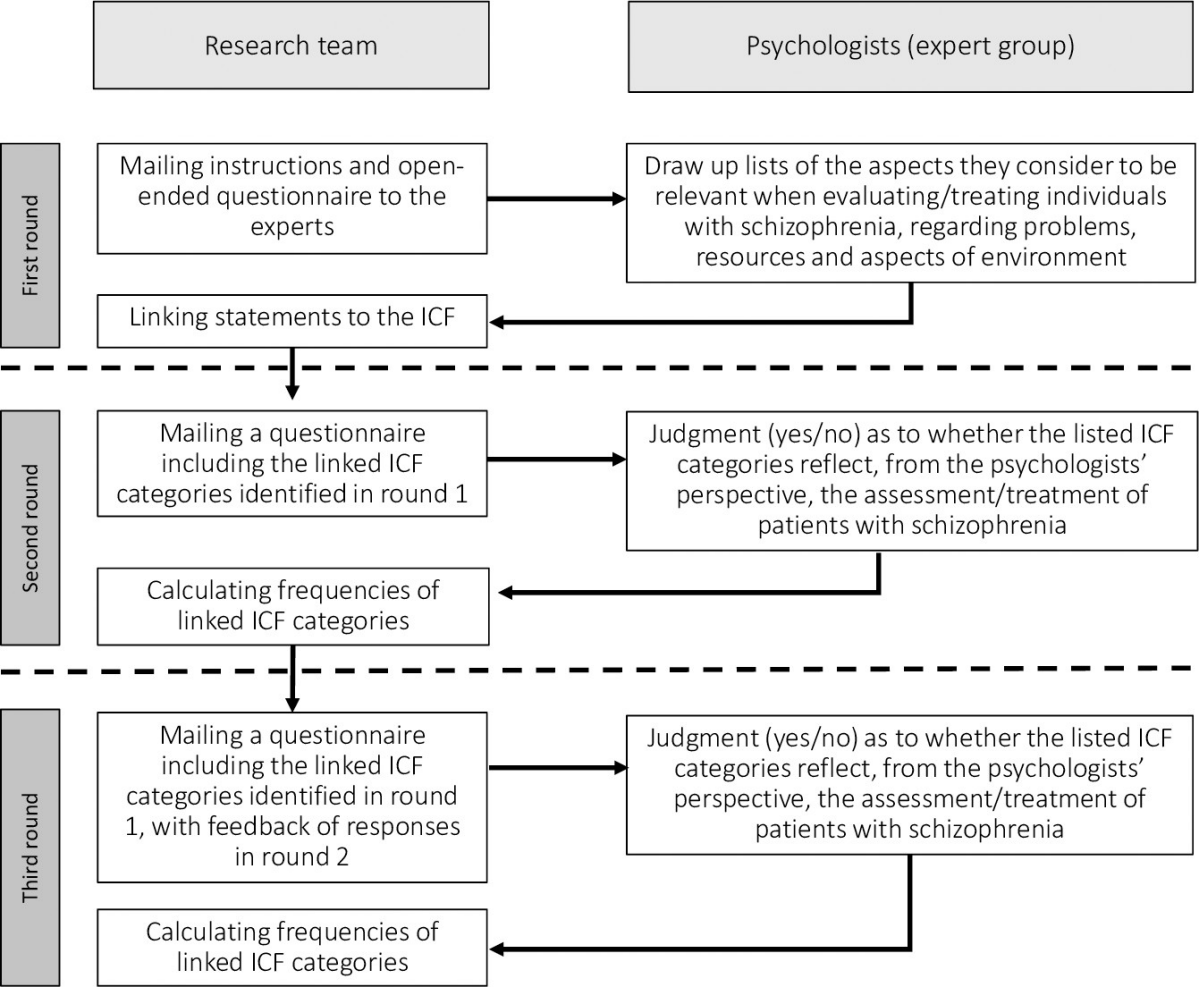
The delphi process.

With the aim of avoiding language barriers and encouraging participation by experts from different world regions, the study was conducted in five languages (Chinese, English, French, Russian, and Spanish). The survey materials were independently translated and supervised by at least two native speakers. The Delphi process is shown in Fig 2. Data were collected between March and June 2017, with participants being allowed two weeks to respond in each round.

Responses in the first Delphi round were logged using an online survey system (www.qualtrics.com). Participants were sent an e-mail with a link to the survey homepage and instructions (i.e., to list all the aspects they considered to be relevant when assessing and/or treating individuals with schizophrenia). To help them with this survey they were asked to consider six open-ended questions that covered all four components of the ICF-CS; the *Environmental factors* component was divided into supportive and hindering factors (survey questions can be consulted in S1 Text). The expected completion time for each survey round was about 15 minutes.

The responses gathered in the first round were then linked to ICF categories using established ICF linking rules [16,17]. All categories reported by at least 5% of the experts were listed and presented to the panel in the second Delphi round. Specifically, all the panelists who had responded in the first round were sent a list of the selected ICF categories linked to the responses of all participants, as well as a list of the categories proposed for *Personal factors*, along with their respective definitions. The categories included in the ICF-CSs for schizophrenia were also listed. For each category, they were asked to indicate whether it was relevant from their perspective as a psychologist to the assessment and/or treatment of individuals with schizophrenia. They were reminded that the aim was to obtain a final list that was both short enough to be applicable in clinical practice and sufficiently comprehensive to cover the most important needs of people with schizophrenia. Participants in the third round were asked to evaluate the same list of categories again, this time taking into account the feedback they were sent concerning the responses of the panel and their own previous responses.

### Linking

**Fig 3:**
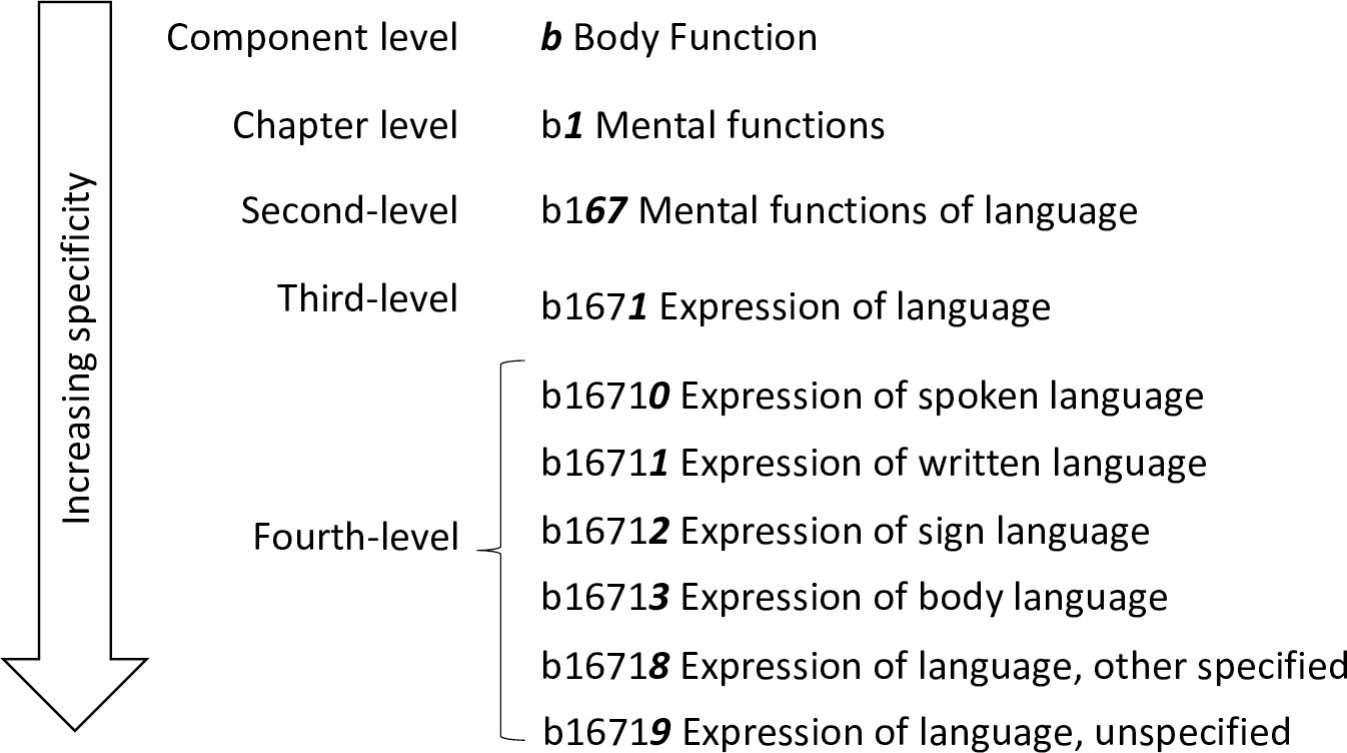
Hierarchical structure of the ICF, exemplified by category ‘b1671 Expression of language’.

All components of the ICF, except *Personal factors*, are organized hierarchically in an exhaustive list of categories (see Fig 3). Third- and fourth-level categories are more specific than second-level categories, and they share the attributes of the second-level category with which they are associated. Therefore, their use implies that the corresponding second-level category is applicable.

Two health professionals with experience of treating persons with schizophrenia and trained in the use of the ICF independently linked all responses from the first Delphi round to the corresponding ICF categories. For instance, if the reported problem was ‘executive dysfunction’, the concept ‘executive function’ was extracted and assigned to the ICF category *b164 Higher-level cognitive functions*. Any disagreements between the two independent coders were reviewed and discussed by two other health professionals with the aim of achieving consensus.

*Personal factors* were defined as the particular background of an individual’s life and living situation (e.g., age) [18]. Personal traits that constitute a premorbid predisposition of individuals and which affect how they cope with their illness were considered as *Personal factors*, whereas personality traits that are altered due to the illness were coded under category *b126* of *Body functions*. As *Personal factors* are not currently categorized in the ICF, they do not feature in the ICF-CS for schizophrenia. However, as they are relevant to assessment and intervention planning, concepts related to *Personal factors* were summarized and considered in rounds two and three of the Delphi study. The proposed categorization of *Personal factors* was developed by consensus among three psychologists (L.N., M.B., G.G.) based on previously proposed categorizations of personal factors [14,18,19] and on the experts’ responses to the question about personal factors.

### Data analysis

We calculated descriptive statistics for the sociodemographic characteristics of participants and the frequencies of ICF categories. In order to be able to compare our findings with the ICF-CSs for schizophrenia, which comprise solely second-level categories, all third- and fourth-level categories identified in the Delphi process were aggregated to their corresponding second-level category.

Based on previous studies [14,20], consensus was defined as agreement among at least 75% of participants. Inter-coder reliability was assessed by calculating the delta statistic and 95% confidence intervals (95% CI) [21]. In order to facilitate comparison with previous studies that use the kappa index, we also calculated this statistic and its 95% CI [22].

The categories for which there was agreement in the third round were compared with the categories included in both the Brief and Comprehensive ICF-CSs.

## Results

### Linking process

From the experts’ answers in round one, a total of 7,526 concepts were extracted and linked to 412 ICF categories (219 second-level, 189 third-level, and 4 fourth-level). Fifty-three categories were proposed for the *Personal factors* identified. Aggregation of third- and fourth-level categories to their corresponding second-level category yielded a list of 223 second-level ICF categories. Those ICF categories and *Personal factors* that were reported by less than 5% of the experts (98 ICF categories and 20 personal factors) were excluded from the second round; ICF categories coded as ‘other specified’ or ‘unspecified’ at the second-level (*n* = 11 ICF categories) were also excluded. This meant that in round two, the panel had to consider a list of 114 second-level ICF categories and 33 *Personal factors*. In the third round, consensus (i.e., agreement of at least 75%) was reached for 76 ICF categories and 28 *Personal factors*. Data regarding the categories presented to experts in rounds two and three and the degree of consensus reached are shown in the first two rows of Table 2. Applying the delta statistic method, a general index of .90 [95% CI: .89 - .91] was obtained, indicating that 90% of agreements were not due to chance. The kappa coefficient for the linking process was .90 [95% CI: .88 - .92].

**Table 2:** Absolute frequencies of second-level ICF categories for which consensus was reached and comparison with the categories included in the Comprehensive ICF-CS for schizophrenia.

Number of categories	ICF components				
	Body functions	Body structures	Activities and Participation	Environmental factors	Total
No. of categories presented to experts in the second and third rounds (n)	19	7	51	37	114
No. of categories for which consensus was reached (n)	14	1	32	29	76
No. of categories in the ICF-CS for schizophrenia (n)	17	0	48	32	97
No. of categories from the ICF-CS for which consensus was reached (n)	13	0	32	28	73

### Correspondence between panel responses and the ICF core sets for schizophrenia

Agreement of 75% or higher was reached for 75.3% of the categories included in the Comprehensive ICF-CS for schizophrenia and for all the categories in the Brief version. Therefore, the following analysis refers solely to the Comprehensive ICF-CS. A summary of the results is shown in the third and fourth row of Table 2. More detail regarding the categories listed by the experts and the corresponding percentage analyses is provided in S1–S5 Tables. Table 3 lists the categories that did not match in the two sets of data (the set of categories included in the ICF-CS for schizophrenia and the set of categories that reached consensus).

**Table 3:** Categories that did not match in the two sets of data.

	ICF Component	ICF category	Percentage of agreement (%)^a^
Categories for which consensus was reached but that do not feature in the Comprehensive ICF-CS	Body functions	b126 Temperament and personality functions	77
	Body structures	s110 Structure of brain	90
	Environmental factors	e135 Products and technology for employment	76
Categories from the Comprehensive ICF-CS for which consensus was not reached	Body functions	b330 Fluency and rhythm of speech functions	66
		b530 Weight maintenance functions	57
		b765 Involuntary movement functions	55
		b640 Sexual functions	52
	Activities and Participation	d855 Non-remunerative employment	74
		d630 Preparing meals	73
		d640 Doing housework	72
		d660 Assisting others	72
		d840 Apprenticeship (work preparation)	72
		d650 Caring for household objects	66
		d950 Political life and citizenship	64
		d475 Driving	51
		d510 Washing oneself	47
		d540 Dressing	47
		d166 Reading	42
		d470 Using transportation	42
		d210 Undertaking a single task	40
		d330 Speaking	39
		d930 Religion and spirituality	39
		d860 Basic economic transactions	38
	Environmental factors	e130 Products and technology for education	74
		e330 People in positions of authority	74
		e555 Associations and organizational services, systems, and policies	74
		e545 Civil protection services, systems, and policies	72

^a^ Percentage of participants who considered the respective ICF category as relevant in the third round.

With respect to the *Body functions* component, an agreement of 75% or higher was achieved for 14 categories. Of these, only one (*b126 Temperament and personality functions*) does not feature in the ICF-CS for schizophrenia. Four of the 17 categories that are included in the ICF-CS for schizophrenia (*b330 Fluency and rhythm of speech functions*, *b530 Weight maintenance functions*, *b640 Sexual functions*, *and b765 Involuntary movement functions*) did not achieve consensus in the Delphi study (see S1 Table for more details).

Regarding the *Body structures* component, the ICF-CS for schizophrenia does not contain any category from this component. However, one of its categories (*s110 Structure of brain*) reached an agreement of 90% in the Delphi study (for more details, see S2 Table). With respect to the *Activities and Participation* component, all the categories that reached consensus (*n* = 32) form part of the ICF-CS for schizophrenia. Sixteen categories from this component that are included in the ICF-CS for schizophrenia did not yield consensus (see S3 Table for more information).

Twenty-nine categories from the *Environmental factors* component yielded agreement of at least 75%, and only one of them (*e135 Products and technology for employment*) is not included in the ICF-CS for schizophrenia. Four categories from this component that do feature in the ICF-CS for schizophrenia did not reach consensus in the Delphi study (see S4 Table).

In summary, only three of the 76 categories that yielded an agreement of at least 75% do not feature in the Comprehensive ICF-CS for schizophrenia. Twenty-four categories that form part of the ICF-CS did not achieve consensus among the experts. Regarding *Personal factors*, which are not classified in the ICF, 33 concepts were presented to the experts, and 28 of these yielded consensus (see S5 Table).

## Discussion

This validation study highlights the functioning-related issues that psychologists encounter in their work with individuals with schizophrenia and considers the extent to which these aspects are covered by the ICF Core Sets for schizophrenia. All categories included in the Brief ICF-CS for schizophrenia were selected by 75% or more of participating experts, thus supporting the relevance of the categories that form this ICF-CS. We will therefore focus on comparing our results with the categories featured in the Comprehensive ICF-CS for schizophrenia. As many of the categories listed in that Core Set were considered important by more than half the experts but did not reach the threshold for consensus (75% agreement), the results are discussed by considering categories that were clearly excluded (50% or less of agreement), those whose relevance appears to be ambiguous (between 50% and 75% of agreement), and those for which there was consensus (75% or more agreement).

Concerning the *Body functions* component, all the categories that yielded consensus belong to chapter *b1 Mental functions*. Some of the categories that achieved higher consensus refer to cognitive functions, such as *b164 Higher-level cognitive functions*. This area is one of the main targets of psychological interventions such as cognitive remediation therapy (CRT), which aims to improve neurocognition and other functional outcomes in individuals with schizophrenia [23]. Psychological interventions also address other categories that were associated with high agreement, namely psychosocial functions (*b122 Global psychosocial functions* [24]), functions affected by negative symptoms (e.g., *b130 Energy and drive functions* and *b152 Emotional functions* [25,26]), and classical symptoms in schizophrenia such as delusions and hallucinations (e.g. *b156 Perceptual functions* [27]). These results differ slightly from those obtained from the perspective of psychiatrists [14]. Although psychiatrists highlighted the importance of many categories from chapter *b1 Mental functions*, they also emphasized other categories from the *Body functions* component, such as *b530 Weight maintenance functions* or *b765 Involuntary movement functions*. This is consistent with the more biomedical perspective of psychiatrists.

Only one of the categories from the *Body functions* component (*b126 Temperament and personality functions*) that reached an agreement of at least 75% is not included in the ICF-CS for schizophrenia. As this category also reached consensus in the validation study from the perspective of psychiatrists it clearly reflects a problem area for these patients [28,29], and therefore its exclusion from the ICF-CS for schizophrenia should be reconsidered. Four categories from the *Body functions* component of the ICF-CS (i.e., *b330 Fluency and rhythm of speech functions*, *b530 Weight maintenance functions*, *b640 Sexual functions*, and *b765 Involuntary movement functions*) did not achieve consensus in the Delphi study but were considered important by more than half the experts. This suggests that these categories are relevant to the assessment of and intervention with persons with schizophrenia, but that they may not be the most common target of psychologists’ interventions, which focus primarily on mental rather than other body functions [23]. In fact, these functions are mainly assessed by other professionals, such as endocrinologists (weight maintenance) or physiotherapists (movement abnormalities).

Although no category from the *Body structures* component is currently included in the ICF-CS for schizophrenia, 90% of the psychologists agreed that brain structure (*s110 Structure of brain*) is an essential aspect to consider when treating individuals with schizophrenia. The relevance of this category was likewise noted in the Delphi study from the perspective of psychiatrists [14], where agreement was even higher (97%). The literature also supports the idea that the brain is the main altered structure in this illness and it is considered to be the basis of other dysfunctions such as neuropsychological impairment [30]. There is also evidence that psychological interventions produce changes in brain structure and its functioning [31], with this being the goal of interventions such as cognitive remediation. Thus, from the perspective of psychologists, inclusion of this category in the ICF-CS for schizophrenia should be considered.

The component with the largest number of categories achieving consensus was *Activities and Participation*. These categories covered all its chapters and focused especially on learning and applying knowledge (e.g., *d160 Focusing attention*), interpersonal interactions (e.g., *d720 Complex interpersonal interactions*), and major life areas such as education (e.g., *d830 Higher education*) and employment (e.g., *d845 Acquiring*, *keeping and terminating a job*). Once again, these results are consistent with those obtained in the validation of the ICF-CS for schizophrenia from the perspective of psychiatrists. All categories of the *Activities and Participation* component for which consensus was reached are listed in the ICF-CS for schizophrenia. This reflects the fact that schizophrenia has a major impact on everyday functioning in all these areas, and illustrates why the main long-term therapeutic goals in the psychological treatment of these individuals are not limited to specific symptoms, but rather focus on improving patients’ psychosocial functioning [32,33]. Sixteen categories that are included in the *Activities and Participation* component of the Comprehensive ICF-CS for schizophrenia were initially referred to by many of our experts but did not reach the threshold for consensus. Of these, the ambiguous categories (i.e., those selected by more than 50% but less than 75% of the expert panel) mainly belong to chapter d6 Domestic life (e.g., *d640 Doing housework*) or are related to employment (e.g., *d855 Non-remunerative employment*). It is worth noting that these categories did yield agreement of 75% or higher in the Delphi study from the perspective of psychiatrists, thus highlighting how different professional views may complement one another. The Comprehensive ICF-CS categories that were selected by fewer than 50% of psychologists mainly referred to simple activities such as *d210 Undertaking a single task* and *d330 Speaking*, whereas consensus was achieved for the equivalent more complex categories (e.g., *d220 Undertaking multiple tasks*). These results offer a more positive view of the abilities of people with schizophrenia, since it suggests that their difficulties mainly depend on the complexity of the task.

As in the previous study from the perspective of psychiatrists, the component with the second highest number of categories showing agreement of at least 75% was *Environmental factors*. The agreed-upon categories especially concerned support and relationships (e.g., *e320 Friends*), attitudes (*e410 Individual attitudes of immediate family members*), and the accessibility of health services (*e580 Health services*, *systems*, *and policies*). These results suggest that psychologists ascribe considerable importance to the impact of environmental factors on the functioning of a person with schizophrenia, a point already made by other authors [34,35]. Of the 29 categories from this component that yielded consensus in the Delphi study, only one (i.e., *e135 Products and technology for employment*) is not included in the ICF-CS for schizophrenia. This category belongs to chapter *e1 Products and Technology*, and it should be noted that the ICF-CS for schizophrenia already contains four categories from the same chapter (i.e., *e110 Products or substances for personal consumption*, *e125 Products and technology for communication*, *e130 Products and technology for education* and *e165 Assets*). Given that an ICF-CS needs to be as short as possible, this domain may already be sufficiently covered by these four categories. Four categories from the *Environmental factors* component of the ICF-CS for schizophrenia did not achieve consensus but were selected by more than 50% of the experts surveyed. This suggests that these categories (e.g., *e555 Associations and organizational services*, *systems*, *and policies*) may be relevant to the assessment and treatment of individuals with schizophrenia, but that they are not primary targets of psychological intervention. Once again, these categories did yield agreement of at least 75% in the Delphi study from the perspective of psychiatrists, underlining the importance of analyzing functionality from a multidisciplinary point of view.

Concerning the *Personal factors* component, we drew up a proposed list of 33 personal factors, 28 of which achieved consensus in the third Delphi round. This level of agreement supports the relevance of personal factors to the assessment and treatment of individuals with schizophrenia. Personal factors, such as resilience [36,37], premorbid cognitive skills [38], premorbid social skills [39], personal history and biography [40], premorbid drug use and lifestyle [41], and premorbid personality [42] have been considered to influence how people with schizophrenia cope with their illness. Most of the categories that psychologists regarded as important coincide with those identified in the validation study from the perspective of psychiatrists [14], suggesting that the proposed list of *Personal factors* captures the aspects that merit particular consideration in this population. In light of these results, it would be useful if the ICF included comprehensive specifications of ‘Personal factors’, or at least a list of such factors, so as to enable more systematic reporting of the personal factors that influence functioning and health and to further stimulate research in this important area [43].

Twenty-four categories that feature in the ICF-CS for schizophrenia did not achieve agreement of 75% in the present Delphi study. This is likely due to the multidisciplinary approach that was used to develop this ICF-CS, which aims to cover the main intervention targets not merely of a specific professional group (in this case, psychologists) but of all health professionals involved in the treatment of individuals with schizophrenia [11].

A particular strength of the present study is that the panel of experts comprised 175 psychologists from 46 countries covering all six WHO regions. Such a large sample is not common in this kind of study [44,45]. Furthermore, all the experts surveyed had considerable experience (54.7% with 10 or more years) in the treatment of patients with schizophrenia, both acute and chronic and from both rural and urban settings. Another strength of the study is that participation was possible in any of five languages, and this is likely to have been a key factor in achieving such a multicultural and multinational representation. It should also be noted that the response rate across rounds one to three was 78%, considerably higher than the mean across rounds of 50% that is reported in the literature [46]. The primary limitation of the study concerns the representativeness of the panel of experts. Although psychologists from all over the world took part, the Eastern Mediterranean, Western Pacific, and African WHO regions were under-represented, and this may limit the external validity of our results. Possible reasons for this under-representation include limited internet access and lower numbers of psychologists in these regions.

To conclude, the results of this study provide strong support for the content validity of the Comprehensive ICF-CSs for schizophrenia as they were obtained by surveying psychologists from all six WHO regions. Of the ICF categories that were selected by at least 75% of experts in the Delphi study, 96% feature in the Comprehensive ICF-CS for schizophrenia. Consensus was achieved for 75.3% of the ICF categories included in the Comprehensive ICF-CS, and 100% of those in the Brief ICF-CS. These results are in line with those obtained in the validation study from the perspective of psychiatrists, where all the categories of the Brief ICF-CS and 90% of those in the Comprehensive version yielded consensus. The fact that there are also some differences in emphasis between psychologists and psychiatrists highlights the importance of considering different professional points of view in order to achieve a fuller picture of how functioning is affected in this population. Taken together, these results suggest that the ICF-CSs for schizophrenia provide a clinically relevant framework for organizing information about this health condition. Having a basic set of categories that addresses a particular patient population at different stages of an illness and that helps both to improve communication within multi-professional teams and to guide the management and treatment of patients by different health professionals is important for ensuring optimal care [47]. The ICF-CSs for schizophrenia can be used as a standard set of ICF categories to facilitate the assessment of functioning in real-life clinical practice by using the ICF qualifiers, which are codes used to record the extent of functioning or disability in a domain or category, or the extent to which an environmental factor is a facilitator or barrier. Importantly, improvement and decline in aspects of functioning can be displayed in a functioning profile over the course of treatment or over the life span. The ICF-CSs for schizophrenia may also be used as a framework for analyzing the content of patient-reported outcome measures or to inform instrument developers about what needs to be included in tools designed to assess the functioning of persons with schizophrenia. Further validation studies from the perspective of other professionals (i.e., nursing, occupational therapy, social work, and physiotherapy) are now needed in order to complement the present findings and to move a step closer towards a definitive version of the ICF-CS for schizophrenia.

## Supporting information

S1 Text Survey questions (round 1). (DOCX)

S2 Text Acknowledgments. (DOCX)

S1 Table Body functions component. (DOCX)

S2 Table Body structures component. (DOCX)

S3 Table Activities and participation component. (DOCX)

S4 Table Environmental factors component. (DOCX)

S5 Table Personal factors component (proposed categories). (DOCX)
