# Supplementary material for: Functioning in schizophrenia from the perspective of psychologists: A worldwide study
Source: PLoS One. 2019 Jun 6;14(6):e0217936. doi: 10.1371/journal.pone.0217936 (PMC6553782; doi:10.1371/journal.pone.0217936)
Supplement: S1 Text — (DOCX) [file pone.0217936.s001.docx]

**S1 Text. Survey questions (round 1).**

- If you think about the **body** and **mind** of individuals with schizophrenia, which problems do you think are relevant for them (which **functions** are affected)?
- If you think about the **body** **parts** of individuals with schizophrenia, in which parts do you think they show problems?
- If you think about the **daily activities** of individuals with schizophrenia, in which ones do you think they have problems?
- If you think about the **environment** and the **living** **conditions** of individuals with schizophrenia, what do you think helps them (that is a **facilitator** for them)?
- If you think about the **environment** and the **living** **conditions** of individuals with schizophrenia, what do you think that hinders them (that is a **barrier** or obstacle for them)?
- If you think about individuals with schizophrenia, which **personal** **characteristics** do you think are important to cope their illness?
