# Supplementary material for: Functioning in schizophrenia from the perspective of psychologists: A worldwide study
Source: PLoS One. 2019 Jun 6;14(6):e0217936. doi: 10.1371/journal.pone.0217936 (PMC6553782; doi:10.1371/journal.pone.0217936)
Supplement: S2 Text — (DOCX) [file pone.0217936.s002.docx]

**S2 Text. Acknowledgments.**

We are grateful to all the experts for their invaluable contribution and the time they invested in responding to the survey. Participants who agreed to be acknowledged in the publication are listed in surname alphabetical order:

F. Afzal, K. Allott, I. Alvarez Tomas, S. Azzali, D. Barch, M. Bechi, M. Bell, M. H. Bin Jamil, I. Birulés, C. Bouvet, J. J. Brofman Epelbaum, L. Byrne, C. Cardoso, X. Castellano, M. Chadzynska, C. Chalkia, V. Chambon, V. Cheung, I. Chrysopoulos, J. Cid, L. Cowley, L. Cremer, M. L. de Bustamante Simas, G. de Souza Lima Brito, C. Demarco, R. Dresdner, C. Duboc, L. Dumitrache, A. Escanilla, G. Escuder, M. Eskin, V. Fenekou, L. Fiorentini, A. Foix, A. Fresan, N. L. Gaines, G. Garrido García, A. M. Gaviria Gómez, V. V. Gijs, L. Glenthøj, J. Gomar, E. Gonzalez-Agua, J. Gottlieb, P. Grandón, E. Grasa, M. Green, K. Grimen, A. Gupta, N. Idrees, I. Incorvaia, Z. Iqbal, L. Irarrázaval, V. O. Jafar, I. J. Jahan, K. Jindal, J. Johannesen, H. Joppich, T. Kahwemba, I. Karaoulani, A. Karistinos, S. Kewming, M. Khader, S. Khodarahimi, A. Kikuchi, D. Kirla, J. Krishnan, N. Kumar, D. Kumar, J. Lee, B. Leonhardt, M. Linke-Jankowska, R. Loewy, H. Long, A. Lucas Hoff Dannevang, J. Luke, M. Lakshmi, A. Mahato, C. Manolatos, D. Mark, E. F. Martínez Rivas, S. A. Mathews, C. McEnery, Z. I. Mohammad, V. Montesano, S. J. Munjanadan, J. Muthee, R. Naranjo Valentín, A. Naumovska, M. Nieznański, J. Noel, I. Nowak, D. Núñez, Y. Olando, G. Oliveira, P. O. O'Reilly, A. Palli, E. J. Pedrero-Pérez, J. Pena-Garijo, S. Perona-Garcelán, D. Perumal, E. Petkari, S. A. Philip, G. Poelke, M. Poletti, A. Polkovnikova-Wamoto, S. Ramachandran, L. Ramos, M. M. Rashad, M. Rizwan, M. Rodriguez, S. Saldivia, N. Schluep, M. Segura, E. Smyth, R. Stavroula, N. Studzinski, K. Subotnik, R. Talwar, N. Thomas, T. Torralba, W. N. Utami, M. Valencia, D. R. Valentino, S. Vallath, L. Valmaggia, A. J. Vázquez Morejón, J. Vehil, D. Velligan, J. Ventura, L. P. Vrklevski, S. Williams, W. Wong, T. Wykes, H. Yazbek, N. Yee, A. Zia & С. Бабин.
