# Supplementary material for: Functioning in schizophrenia from the perspective of psychologists: A worldwide study
Source: PLoS One. 2019 Jun 6;14(6):e0217936. doi: 10.1371/journal.pone.0217936 (PMC6553782; doi:10.1371/journal.pone.0217936)
Supplement: S1 Table — (DOCX) [file pone.0217936.s003.docx]

**S1 Table. Body functions component.**

| ICF code | ICF category | Percentage (%)^a^ | Consensus among experts | Included in ICF Core Set |
| --- | --- | --- | --- | --- |
| b114 | Orientation functions | 79 | x | x |
| b117 | Intellectual functions | 76 | x | x |
| b122 | Global psychosocial functions | 96 | x | x^b^ |
| b126 | **Temperament and personality functions** | 77 | x |  |
| b130 | Energy and drive functions | 99 | x | x^b^ |
| b134 | Sleep functions | 89 | x | x |
| b140 | Attention functions | 98 | x | x^b^ |
| b144 | Memory functions | 88 | x | x |
| b147 | Psychomotor functions | 96 | x | x |
| b152 | Emotional functions | 99 | x | x^b^ |
| b156 | Perceptual functions | 98 | x | x^b^ |
| b160 | Thought functions | 100 | x | x^b^ |
| b164 | Higher-level cognitive functions | 100 | x | x^b^ |
| b180 | Experience of self and time functions | 92 | x | x^b^ |
| b330 | *Fluency and rhythm of speech functions* | 66 |  | x |
| b530 | *Weight maintenance functions* | 57 |  | x |
| b640 | *Sexual functions* | 52 |  | x |
| b760 | Control of voluntary movement functions | 45 |  |  |
| b765 | *Involuntary movement functions* | 55 |  | x |

Abbreviations: ICF, International Classification of Functioning, Disability and Health.

*Italic text***:** Categories from the ICF-CS for schizophrenia for which consensus was not reached in the third round of the Delphi study.

**Bold text:** Categories for which consensus was reached in the third Delphi round but that do not feature in the ICF-CS for schizophrenia.

^a^ Percentage of participants who considered the respective ICF category as relevant in the third round (n=137).

^b^ Categories included in the Brief ICF-CS for schizophrenia.
