# Supplementary material for: Functioning in schizophrenia from the perspective of psychologists: A worldwide study
Source: PLoS One. 2019 Jun 6;14(6):e0217936. doi: 10.1371/journal.pone.0217936 (PMC6553782; doi:10.1371/journal.pone.0217936)
Supplement: S2 Table — (DOCX) [file pone.0217936.s004.docx]

**S2 Table. Body structures component.**

| ICF code | ICF category | Percentage (%)^a^ | Consensus among experts | Included in ICF Core Set |
| --- | --- | --- | --- | --- |
| s110 | **Structure of brain** | 90 | x |  |
| s320 | Structure of mouth | 10 |  |  |
| s410 | Structure of cardiovascular system | 17 |  |  |
| s430 | Structure of respiratory system | 12 |  |  |
| s580 | Structure of endocrine glands | 10 |  |  |
| s710 | Structure of head and neck region | 9 |  |  |
| s730 | Structure of upper extremity | 6 |  |  |
| s750 | Structure of lower extremity | 8 |  |  |

Abbreviations: ICF, International Classification of Functioning, Disability and Health.

**Bold text:** Categories for which consensus was reached in the third Delphi round but that do not feature in the ICF-CS for schizophrenia.

^a^ Percentage of participants who considered the respective ICF category as relevant in the third round (n=137).
