# Supplementary material for: Functioning in schizophrenia from the perspective of psychologists: A worldwide study
Source: PLoS One. 2019 Jun 6;14(6):e0217936. doi: 10.1371/journal.pone.0217936 (PMC6553782; doi:10.1371/journal.pone.0217936)
Supplement: S3 Table — (DOCX) [file pone.0217936.s005.docx]

**S3 Table. Activities and Participation component.**

| ICF code | ICF category | Percentage (%)^a^ | Consensus among experts | Included in ICF Core Set |
| --- | --- | --- | --- | --- |
| d155 | Acquiring skills | 75 | x | x^b^ |
| d160 | Focusing attention | 99 | x | x |
| d163 | Thinking | 94 | x | x |
| d166 | *Reading* | 42 |  | x |
| d175 | Solving problems | 96 | x | x^b^ |
| d177 | Making decisions | 93 | x | x |
| d210 | *Undertaking a single task* | 40 |  | x |
| d220 | Undertaking multiple tasks | 96 | x | x |
| d230 | Carrying out daily routine | 94 | x | x^b^ |
| d240 | Handling stress and other psychological demands | 98 | x | x^b^ |
| d310 | Communicating with - receiving - spoken messages | 77 | x | x |
| d315 | Communicating with - receiving - nonverbal messages | 88 | x | x |
| d330 | *Speaking* | 39 |  | x |
| d335 | Producing nonverbal messages | 77 | x | x |
| d350 | Conversation | 93 | x | x |
| d470 | *Using transportation* | 42 |  | x |
| d475 | *Driving* | 51 |  | x |
| d510 | *Washing oneself* | 47 |  | x |
| d520 | Caring for body parts | 83 | x | x |
| d530 | Toileting | 38 |  |  |
| d540 | *Dressing* | 47 |  | x |
| d550 | Eating | 28 |  |  |
| d570 | Looking after one's health | 96 | x | x^b^ |
| d610 | Acquiring a place to live | 91 | x | x |
| d620 | Acquisition of goods and services | 77 | x | x |
| d630 | *Preparing meals* | 73 |  | x |
| d640 | *Doing housework* | 72 |  | x |
| d650 | *Caring for household objects* | 66 |  | x |
| d660 | *Assisting others* | 72 |  | x |
| d710 | Basic interpersonal interactions | 82 | x | x^b^ |
| d720 | Complex interpersonal interactions | 97 | x | x^b^ |
| d730 | Relating with strangers | 80 | x | x |
| d740 | Formal relationships | 81 | x | x |
| d750 | Informal social relationships | 88 | x | x |
| d760 | Family relationships | 90 | x | x^b^ |
| d770 | Intimate relationships | 92 | x | x |
| d810 | Informal education | 38 |  |  |
| d820 | School education | 89 | x | x |
| d825 | Vocational training | 81 | x | x |
| d830 | Higher education | 85 | x | x |
| d840 | *Apprenticeship (work preparation)* | 72 |  | x |
| d845 | Acquiring, keeping and terminating a job | 96 | x | x^b^ |
| d850 | Remunerative employment | 89 | x | x |
| d855 | *Non-remunerative employment* | 74 |  | x |
| d860 | *Basic economic transactions* | 38 |  | x |
| d865 | Complex economic transactions | 85 | x | x |
| d870 | Economic self-sufficiency | 89 | x | x |
| d910 | Community life | 85 | x | x^b^ |
| d920 | Recreation and leisure | 95 | x | x |
| d930 | *Religion and spirituality* | 39 |  | x |
| d950 | *Political life and citizenship* | 64 |  | x |

Abbreviations: ICF, International Classification of Functioning, Disability, and Health.

*Italic text*: Categories from the ICF-CS for schizophrenia for which consensus was not reached in the third round of the Delphi study.

^a^ Percentage of participants who considered the respective ICF category as relevant in the third round (n=137).

^b^ Categories included in the Brief ICF-CS for schizophrenia.
