# Supplementary material for: Functioning in schizophrenia from the perspective of psychologists: A worldwide study
Source: PLoS One. 2019 Jun 6;14(6):e0217936. doi: 10.1371/journal.pone.0217936 (PMC6553782; doi:10.1371/journal.pone.0217936)
Supplement: S4 Table — (DOCX) [file pone.0217936.s006.docx]

**S4 Table. Environmental factors component.**

| ICF code | ICF category | Percentage (%)^a^ | Consensus among experts | Included in ICF Core Set |
| --- | --- | --- | --- | --- |
| e110 | Products or substances for personal consumption | 96 | x | x |
| e115 | Products and technology for personal use in daily living | 69 |  |  |
| e125 | Products and technology for communication | 82 | x | x |
| e130 | *Products and technology for education* | 74 |  | x |
| e135 | **Products and technology for employment** | 76 | x |  |
| e140 | Products and technology for culture, recreation, and sport | 69 |  |  |
| e165 | Assets | 85 | x | x |
| e250 | Sound | 48 |  |  |
| e310 | Immediate family | 99 | x | x^b^ |
| e315 | Extended family | 91 | x | x |
| e320 | Friends | 98 | x | x |
| e325 | Acquaintances, peers, colleagues, neighbors, and community members | 93 | x | x |
| e330 | *People in positions of authority* | 74 |  | x |
| e340 | Personal care providers and personal assistants | 92 | x | x |
| e355 | Health professionals | 96 | x | x^b^ |
| e360 | Other professionals | 91 | x | x |
| e410 | Individual attitudes of immediate family members | 99 | x | x^b^ |
| e415 | Individual attitudes of extended family members | 78 | x | x |
| e420 | Individual attitudes of friends | 93 | x | x |
| e425 | Individual attitudes of acquaintances, peers, colleagues, neighbors, and community members | 89 | x | x |
| e430 | Individual attitudes of people in positions of authority | 79 | x | x |
| e440 | Individual attitudes of personal care providers and personal assistants | 91 | x | x |
| e450 | Individual attitudes of health professionals | 93 | x | x^b^ |
| e455 | Individual attitudes of other professionals | 81 | x | x |
| e460 | Societal attitudes | 91 | x | x^b^ |
| e465 | Social norms, practices, and ideologies | 97 | x | x |
| e525 | Housing services, systems, and policies | 89 | x | x |
| e535 | Communication services, systems, and policies | 68 |  |  |
| e545 | *Civil protection services, systems, and policies* | 72 |  | x |
| e550 | Legal services, systems, and policies | 85 | x | x |
| e555 | *Associations and organizational services, systems, and policies* | 74 |  | x |
| e560 | Media services, systems, and policies | 75 | x | x |
| e570 | Social security services, systems, and policies | 88 | x | x^b^ |
| e575 | General social support services, systems, and policies | 96 | x | x |
| e580 | Health services, systems, and policies | 97 | x | x^b^ |
| e585 | Education and training services, systems, and policies | 90 | x | x |
| e590 | Labor and employment services, systems, and policies | 94 | x | x |

Abbreviations: ICF, International Classification of Functioning, Disability and Health.

*Italic text*: Categories from the ICF-CS for schizophrenia for which consensus was not reached in the third round of the Delphi study.

**Bold text**: Categories for which consensus was reached in the third Delphi round but that do not feature in the ICF-CS for schizophrenia.

^a^ Percentage of participants who considered the respective ICF category as relevant in the third round (n=137).

^b^ Categories included in the Brief ICF-CS for schizophrenia.
