# Supplementary material for: Functioning in schizophrenia from the perspective of psychologists: A worldwide study
Source: PLoS One. 2019 Jun 6;14(6):e0217936. doi: 10.1371/journal.pone.0217936 (PMC6553782; doi:10.1371/journal.pone.0217936)
Supplement: S5 Table — (DOCX) [file pone.0217936.s007.docx]

**S5 Table. Personal factors component (proposed categories).**

| Personal factor^a^ | Percentage^b^ | Consensus Delphi |
| --- | --- | --- |
| ***Habits and lifestyle*** | |  |
| Drug use | 94 | x |
| Lifestyle | 94 | x |
| ***Personality and other psychological characteristics*** | | |
| Agreeableness | 82 | x |
| Attitudes towards health | 95 | x |
| Autonomy | 91 | x |
| Conscientiousness | 75 | x |
| Extraversion | 62 |  |
| Motivation | 94 | x |
| Neuroticism | 85 | x |
| Openness to experience | 82 | x |
| Optimism | 86 | x |
| Personal attitude | 92 | x |
| Personality | 96 | x |
| Resilience | 97 | x |
| Self-awareness | 90 | x |
| Self-esteem | 96 | x |
| Spirituality and religiosity | 64 |  |
| ***Personal skills*** |  |  |
| Cognitive skills | 96 | x |
| Coping skills | 96 | x |
| Intelligence | 86 | x |
| Psychosocial skills | 98 | x |
| ***Sociodemographic variables*** |  |  |
| Age | 81 | x |
| Ethnicity | 34 |  |
| Gender | 42 |  |
| Level of education | 84 | x |
| Living situation | 98 | x |
| Marital status | 76 | x |
| Occupational status | 91 | x |
| Socioeconomic status | 86 | x |
| ***Other personal factors*** |  |  |
| Age at onset | 98 | x |
| Genetics | 69 |  |
| Personal history and biography | 98 | x |
| Premorbid functioning | 86 | x |

^a^ Each proposed personal factor category was accompanied by its definition in the second and third round.

^b^ Percentage of participants who considered each proposed category as relevant in the third round (n=137).
